# MiR-186 inhibited aerobic glycolysis in gastric cancer via HIF-1α regulation

**DOI:** 10.1038/oncsis.2016.35

**Published:** 2016-05-09

**Authors:** L Liu, Y Wang, R Bai, K Yang, Z Tian

**Affiliations:** 1 Department of General Surgery, Shengjing Hospital of China Medical University, Shenyang, People's Republic of China; 2 Department of General Surgery, The Fourth Hospital of China Medical University, Shenyang, People's Republic of China

## Abstract

Deregulation of microRNAs in human malignancies has been well documented, among which microRNA-186 (miR-186) has an antiproliferative role in some carcinomas. Here we demonstrate that low expression of miR-186 facilitates aerobic glycolysis in gastric cancer. MiR-186 suppresses cell proliferation induced by hypoxia inducible factor 1 alpha (HIF-1α) in gastric cancer cell lines MKN45 and SGC7901. Cellular glycolysis, including cellular glucose uptake, lactate, ATP/ADP and NAD+/NADH ratios, are also inhibited by miR-186. The negative regulation of miR-186 on HIF-1α effects its downstream targets, including programmed death ligand 1 and two glycolytic key enzymes, hexokinase 2 and platelet-type phosphofructokinase. The antioncogenic effects of miR-186 are proved by *in vivo* xenograft tumor experiment. The results demonstrate that the miR-186/HIF-1α axis has an antioncogenic role in gastric cancer.

## Introduction

The poorly differentiated gastric adenocarcinoma is associated with bad prognosis and resistance to chemotherapy.^1, 2, 3^ Its rapid progression is largely based on glycolysis for energy metabolism even under ambient oxygen condition, the phenomenon is named as 'Warburg effect'.^4^ The 'Warburg effect' is the most common malignant metabolic feature, which was described by Warburg 90 years ago. It claims that, under aerobic condition, glycolytic fermentation—the procedure that glucose is metabolized to lactic acid, is a major metabolic way of tumor cells.^5^ The Warburg effect-based examination is currently widely used, such as positron emission tomography. Although it is well accepted that the Warburg effect exists in gastric cancer, the driving mechanism of aerobic glycolysis still remains largely unknown. Therefore, searching for the deep mechanism is urged for therapeutic aims.

MicroRNAs form a complex regulatory network of biological processes and participate in life activities, including oncogenesis.^6^ MicroRNA-186 (miR-186) acts as a tumor suppressor in many malignancies.^7, 8, 9^ It could modulate sensitivity to chemotherapeutics.^10^ As a potential therapy target, miR-186 is rarely studied in gastrointestinal carcinoma. In the present study, we seek to determine whether and how aberrant expression of miR-186 effects on gastric adenocarcinoma cell proliferation, apoptosis.

It is a frequent condition that tumor cells are exposed to low nutrient and oxygen condition. Thus, to adapt to the microenviroment, hypoxic-related genes are frequently activated, one of which is hypoxia inducible factor-1 (HIF-1).^11^ Under hypoxia conditions such as tumor center, it is stably expressed. HIF-1α, as the active subunit of HIF-1,^12^ regulates transcription of quite a wide spectrum of target genes in gastric cancer.^13^ It regulates hypoxia adaptation and has an important role in aerobic glycolysis and tumorigenesis.^14, 15^ The metabolic effects such as biomass accumulation and allocation of glucose into ATP require normoxic stabilization of HIF-1α. HIF-1α expression remains as independent prognostic variables for gastric adenocarcinoma.^16^ At present, most of the studies focus on the regulation of downstream genes of HIF-1α instead of HIF-1α its own generation control.^17, 18^

As a target gene of HIF-1α, PD-L1^19^ is a ligand of programmed death protein 1,^20^ a T-cell coinhibitory receptor. PD-L1 has a pivotal role in tumor cells' immune evading, the ability of escaping from the host's immune system. Experts identified that upregulation of PD-L1 is involved in tumorigenesis and poor prognosis in gastric cancer.^21^ As another HIF-1α target, hexokinase is one of the rate-limiting enzymes of glycolytic pathway.^22, 23^ In mammals, there are four subtypes of hexokinase, the type II hexokinase is insulin sensitive and mostly overexpressed in poorly differentiated tumor tissues.^24^ Another rate-limiting enzyme of glycolytic pathway is PFK-1 (phosphofructokinase 1), a putative transcriptional target of HIF-1α. It participates in breast cancer progression.^25^ PFK-1 is a complex tetrameric enzyme and exists in three isoforms: platelet (PFKP), muscle (PFKM), and liver (PFKL), among which PFKP is reported the major isoform in tumor.^26, 27^

In this present study, the *in vitro* and *in vivo* study are carried out. We seek to determine whether and how miR-186/HIF-1α axis could reprogram cellular metabolism and proliferation in gastric adenocarcinoma.

## Results

### MiR-186 was lowly expressed in human gastrocarcinama

**Figure 1:**
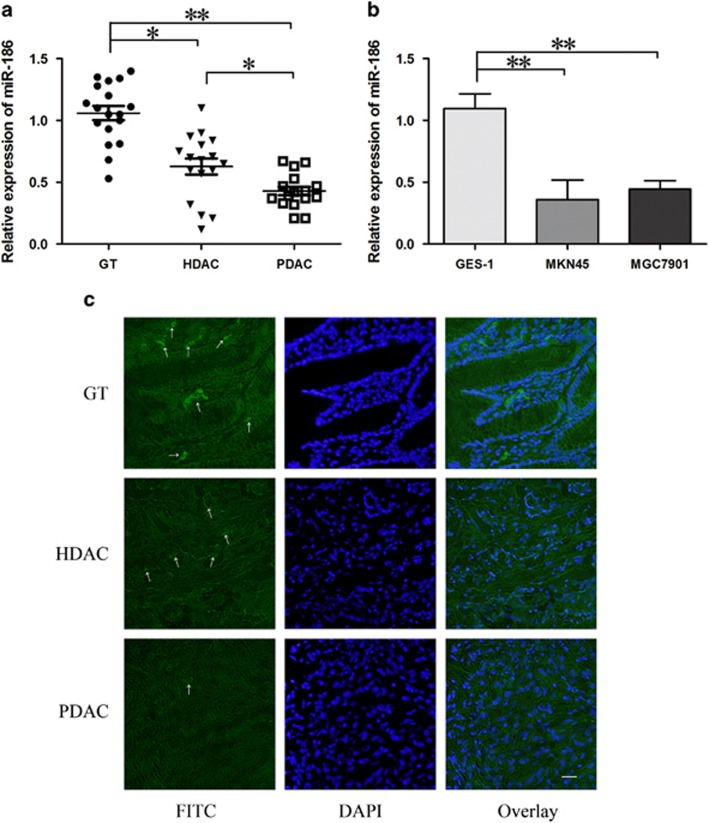
MiR-186 was lowly expressed in human gastric cancer tissues (**a**) and cell lines (**b**). Eighteen cases of normal GT, 17 cases of HDAC tissues, 16 cases of PDAC tissues were obtained from surgical resection in Shengjing Hospital of China Medical University. Expression levels of miR-186 were determined by qRT–PCR in control GT, HDAC and PDAC MKN45 and SGC7901 glioma cells. (**c**) Hybridization *in situ* for miR-186, green fluorescent dots were represented as positive. Data were presented as mean±s.d. from three independent experiments. *P<0.05, **P<0.01.

MiR-186 is not yet reported in gastric cancer tissues and its biological function remains largely unknown. Quantitative real-time PCR (qRT–PCR) was applied to detect miR-186 abundance in normal gastric tissues (GT), highly differentiated gastric adenocarcinoma tissues (HDAC), poorly differentiated gastric adenocarcinoma tissues (PDAC) and cell lines GES-1, MKN45 and SGC7901. The results demonstrated that miR-186 was significantly downregulated in HDAC specimens, it was even lower in PDAC (Figure 1a). MiR-186 content in MKN45 and SGC7901 cells was also lower than in normal gastric mucosa cell line GES-1 (Figure 1b). MiR-186 locations in GT, HDAC and PDAC are shown in Figure 1c. The arrows indicated the spots of miR-138 hybridized. Fluorescence intensity of GT was higher than HDAC and PDAC. The reverse correspondence between miR-186 expression and pathological grading prompted that miR-186 may act as a tumor suppressor in gastric adenocarcinoma.

### MiR-186 inhibited MKN45 and SGC7901 cell proliferation and promoted apoptosis

**Figure 2:**
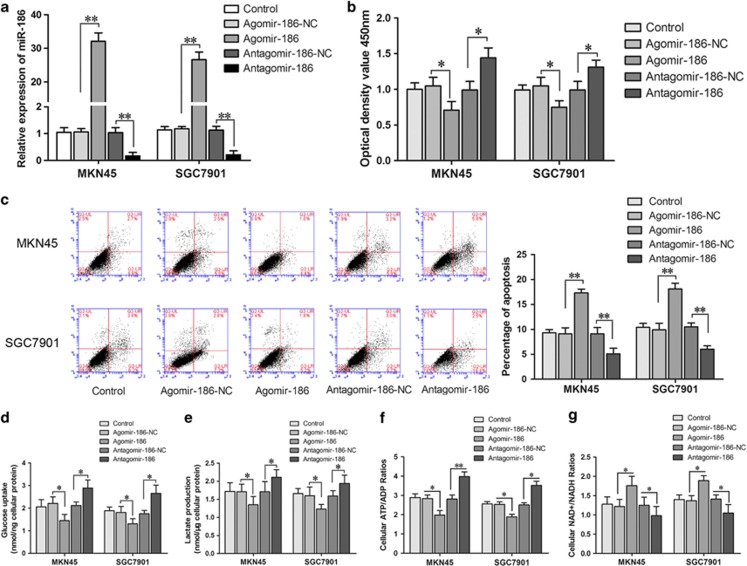
Overexpressed miR-186 inhibited cell proliferation, glucose uptake, lactate accumulation, ATP/ADP ratio, increased apoptosis and NAD+/NADH ratio in MKN45 and SGC7901 cells. (**a**) MiR-186 expression levels were evaluated using qRT–PCR in agomir-186- and antagomir-186-transfected MKN45 and SGC7901 cells. (**b**) CCK8 assays were performed to determine the cell viability of MKN45 and SGC7901 cell lines stably overexpressing or knockdown of miR-186. (**c**) The apoptotic percentages of MKN45 and SGC7901 cells stably overexpressing miR-186 by flow cytometry. LR, early apoptotic cells; UL, necrotic cells; UR, terminal apoptotic cells. (**d**) The glucose uptake assay, (**e**) lactate accumulation, (**f**) ATP/ADP ratio and (**g**) NAD+/NADH ratio of MKN45 and SGC7901 cell lines stably overexpressing or knockdown of miR-186. Data were presented as mean±s.d. from three independent experiments. *P<0.05, **P<0.01.

To further investigate any potential differences at the functional level, miR-186 overexpression and knockdown cell lines were established (Figure 2a). Cells viabilities were inhibited in the agomir-186 groups of both MKN45 and SGC7901 cells, and apoptotic rates were increased (*P*<0.05; Figures 2b and c). Results were just the opposite in the antagomir-186 groups. It demonstrated that miR-186 reliably had an antiproliferative role in MKN45 and SGC7901 cells.

### MiR-186 inhibited glycolysis in MKN45 and SGC7901 cells

MKN45 and SGC7901 cells that overexpressed miR-186 had less intracellular glucose, lactate production and ATP/ADP ratio, besides NAD+/NADH ratios were higher than the agomir-186-NC groups (*P*<0.05). In parallel, the antagomir-186 groups harbored higher intracellular glucose, lactate and ATP/ADP ratio and less NAD+/NADH (Figures 2d–g). The results hinted that miR-186 could inhibit cellular glycolysis steps.

### MiR-186 targeted HIF-1α and inhibited its protein expression

**Figure 3:**
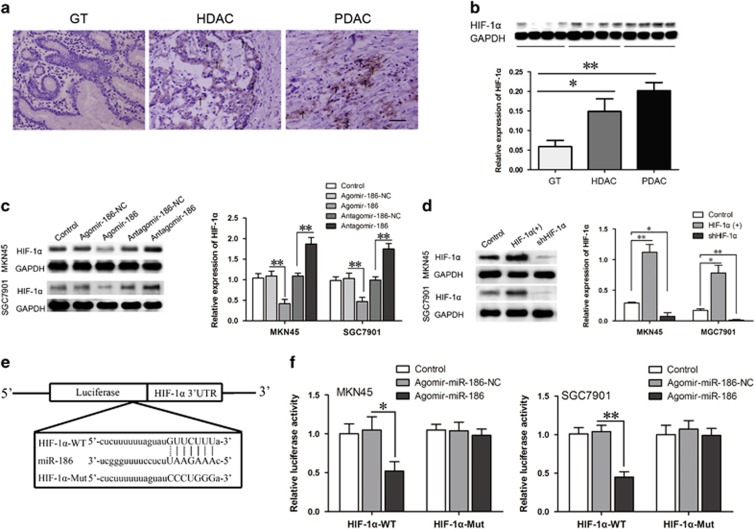
MiR-186 targeted HIF-1α and inhibited its protein expression. (**a**, **b**) Immunohistochemistry assay and western blotting for HIF-1α protein in normal GT and gastric cancer tissues. (**c**, **d**) Western blotting for HIF-1α expression in MKN45 and SGC7901 cell lines stably overexpressing or knockdown of miR-186 (or HIF-1α). (**e**) The predicted miR-186-binding sites in the 3′-UTR region of HIF-1α (HIF-1α-3′UTR-Wt) and the designed mutant sequence (HIF-1α-3′UTR-Mut) are indicated. (**f**) Luciferase assay of miR-186 and HIF-1α was conducted. Relative luciferase activities for the HIF-1α-3′UTR-Wt and HIF-1α-3′UTR-Mut groups. Data are presented as the mean±s.d. *P<0.05, **P<0.01.

HIF-1α drew wide attention recently for its role in tumorgenesis and progression. Overexpression of HIF-1α was identified in gastric cancer, and it was positively correlated with pathological grade and poor prognosis.^28, 29^ Immunohistochemistry assays and western blotting were performed to investigate HIF-1α content in GT, HDAC and PDAC (Figures 3a and b). The results were consistent with previous reports. HIF-1α expression in HDAC was higher than in normal GT; the expression in PDAC was much higher.

Protein expression levels of HIF-1α were analyzed when miR-186 or HIF-1α was overexpressed or knocked down. Compared with the agomir-186-NC group, HIF-1α levels were higher in the agomir-186 groups, whereas lower in the antagomir-186 groups compared with the antagomir-186-NC groups (Figure 3c). HIF-1α introduction resulted in nearly threefold high expression of HIF-1α, while HIF-1α ablation led to obvious reduction of HIF-1α expression (Figure 3d).

To further clarify the precise regulation mechanism of miR-186 to HIF-1α, luciferase reporter assay was conducted (Figures 3e and f). The results showed that the fluorescence intensity was weaker in the group cotransfected with agomir-186 and HIF-1α-3′UTR-WT compared with the control group; however, fluorescence density did not change in the group cotransfected with agomir-186-NC and HIF-1α-3′UTR-WT. In the group cotransfected with agomir-186 and HIF-1α-3′UTR-MUT, there was no change in fluorescence intensity either. The results confirmed that HIF-1α was the direct target of miR-186 in gastric adenocarcinoma.

### MiR-186 inhibited proliferation and glycolysis process and induced apoptosis by targeting HIF-1α-3′UTR in MKN45 and SGC7901 cells

**Figure 4:**
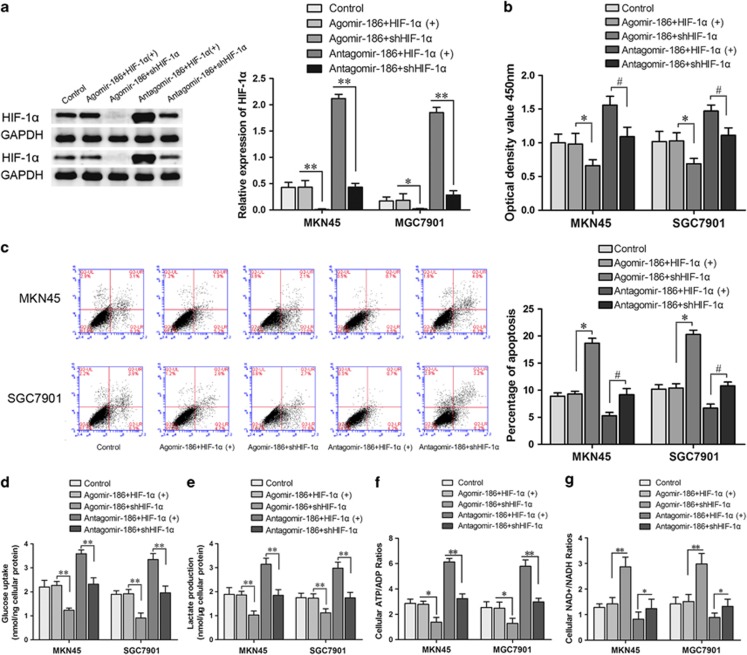
Overexpression of HIF-1α reversed miR-186-induced inhibitory effects on gastric cancer cells. (**a**) Western blotting for HIF-1α expression in MKN45/SGC7901 HIF-1α (+) and shHIF-1α cells transfected with agomir-186 and antagomir-186. (**b**) Overexpression of HIF-1α partly reversed miR-186-induced inhibition of proliferation in MKN45 and SGC7901 cells determined by CCK8 assay. (**c**) Overexpression of HIF-1α partly reversed miR-186-induced apoptosis. Overexpression of HIF-1α partly reversed miR-186-induced (**d**) glucose uptake, (**e**) lactate production, (**f**) cellular ATP/ADP ratio and (**g**) NAD+/NADH ratio in MKN45 and SGC7901 cells. *P<0.05, **P<0.01, ^#^*P*<0.05.

To further investigate whether miR-186 functions through targeting HIF-1α, we transfected agomir-186 and antagomir-186 into HIF-1α stably overexpressed or knockdown cells. The establishment of a stable transfected cell line is described in detail in Materials and methods section. HIF-1α protein expression levels are shown in Figure 4a. In the agomir-186+shHIF-1α groups, HIF-1α expression levels were hard to detect; in the antagomir-186+HIF-1α(+) groups, HIF-1α levels were obviously increased. The results in Figures 4b and c showed that the attenuated cell viability and high cell death induced by agomir-186 could be corrected through HIF-1α overexpression; also, abnormal high proliferation and reduced cell apoptosis caused by antagomir-186 could be weakened by HIF-1α knockdown.

As for glycolysis process, miR-186 overexpression inhibited glucose uptake, lactate and ATP and NADH accumulation, whereas HIF-1α cotransfection rescued the glucose uptake, lactate, ATP and NADH production; miR-186 knockdown upregulated the glucose uptake, lactate and ATP/ADP ratio, meanwhile it downregulated NAD+/NADH; however, HIF-1α knockdown attenuated the process (Figures 4d–g).

### Overexpression of miR-186 downregulated PD-L1 and glycolytic rate-limiting enzyme HK2, PFKP content and activities by inhibiting HIF-1α

**Figure 5:**
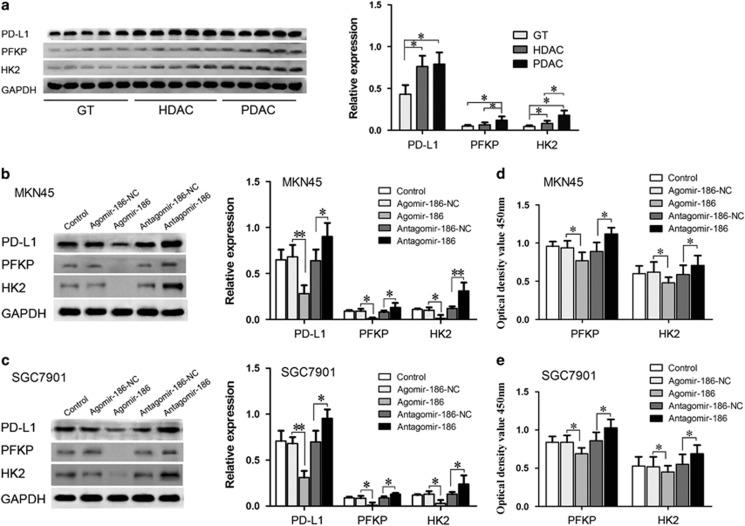
PD-L1, PFKP and HK2 were highly expressed in gastric cancer tissues and their expression levels were inhibited by miR-186. (**a**) PD-L1, PFKP and HK2 expression levels in normal GTs and cancer tissues. (**b**, **c**) PD-L1, PFKP and HK2 expression levels were downregulated by miR-186 in western blotting assays in MKN45 and SGC7901 cell lines. (**d**, **e**) PFKP and HK2 enzyme activities were inhibited by miR-186 introduction. *P<0.05, **P<0.01.

**Figure 6:**
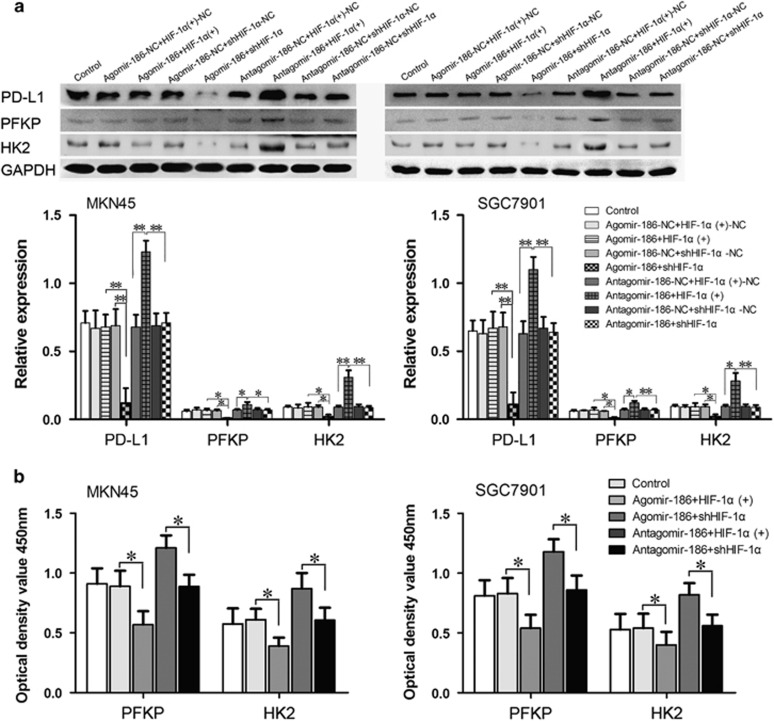
Overexpression of HIF-1α reversed miR-186-induced inhibitory effects on PD-L1, PFKP and HK2 protein abundance and PFK and HK2 activities. (**a**) Overexpression of HIF-1α partly reversed miR-186-induced inhibition on PD-L1, PFKP and HK2 protein expression levels. (**b**) Introduction of HIF-1α partly reversed miR-186-induced inhibition on PFK and HK2 activities. *P<0.05, **P<0.01.

PFKL and PFKM expression levels were in trace amount and hard to detect in the collected specimens and MKN45 and SGC7901 cell lines, so PFKP expression was indicated here to represent PFK amount. PD-L1 HK2 and PFKP expression levels were detected in specimens and cell lines by western blotting (Figure 5a). PD-L1, HK2 and PFKP expression levels (Figures 5b and c) as well as HK2 and PFK activities (Figures 5d and e) were downregulated by miR-186 introduction and were rescued by HIF-1α overexpression (Figures 6a and b). PD-L1, HK2 and PFKP expression levels as well as HK2 and PFK activities were upregulated in the antagomir-186 group and the upregulatory effect was counteracted by HIF-1α knockdown. These results suggested that miR-186 could reduce PD-L1 abundance and HK2 and PFKP expression levels and activities by targeting HIF-1α.

### HIF-1α targeted PFKP and activated promoter transcription

**Figure 7:**
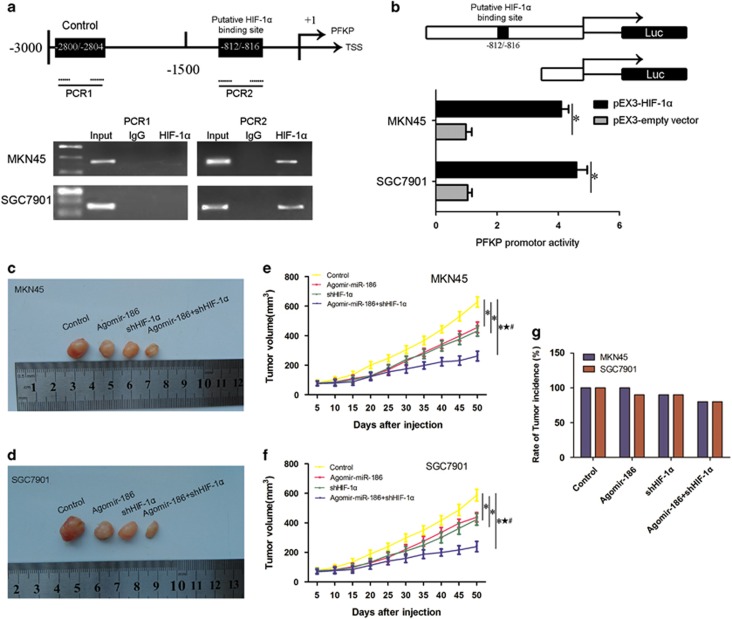
HIF-1α activated the transcription of PFKP and nude mice xenograft experiment for miR-186 and HIF-1α gene effect. (**a**) HIF-1α binded to the HRE in the PFKP promoter region. (**b**) HIF-1α activated the promoter transcription of PFKP. *P<0.05 (**c**–**f**) *In vivo* nude mice xenograft study. The nude mice carrying tumors and tumor sizes from respective groups are represented. (**g**) Percentage of tumor incidence in each group. *P<0.05 vs control group, ^★^*P*<0.05 vs agomir-186 group, ^#^*P*<0.05 vs shHIF-1α group.

By screening the promoter region of PFKP, we found a potential binding region of HIF-1α. In the chromatin immunoprecipitation (ChIP) assay (Figure 7a), the position of the transcription start site was predicted by Database of human Transcription Start Sites. HIF-1α binds 'CACGC' sequence, which is the promoter element of target genes.^30^ Wild-type and putative HIF-1α-binding sites were indicated. Pulled down by anti-HIF-1α antibody, the PCR fragments of PFKP promoter (−812/−816) were detected under normoxia. In samples pulled down by a control immunoglobulin G (IgG) antibody, no PCR fragment was detected. The ChIP assay showed that there was an interaction between HIF-1α and the putative binding site of PFKP. No interaction was observed between HIF-1α and negative control region.

To clarify whether the binding of HIF-1α to PFKP promoter could activate the PFKP transcription in MKN45 and SGC7901 cells, promoter luciferase assays were performed (Figure 7b). Cotransfected with pEX3-HIF-1α, PFKP promoter activities were upregulated by 4.2- and 4.5-fold in MKN45 and SGC7901 cells, respectively. The luciferase assays and ChIP results taken together demonstrated that HIF-1α could bind to the PFKP promoters and upregulate the promoter activities in human MKN45 and SGC7901 cells.

### Overexpression of miR-186 combined with HIF-1α knockdown suppressed tumor growth in nude mice xenograft assay

The results above prompted us to examine the effective mechanism of the miR-186–HIF-1α axis on tumorous growth *in vivo*. Stable cell lines constructed as previously described were inoculated into nude mice, and tumor volumes were monitored as shown in Figures 7c and d. The agomir-186, shHIF-1α and agomir-186 combined with shHIF-1α groups all generated smaller tumors than the control group in both MKN45 and SGC7901 cells. Agomir-186 combined with shHIF-1α group produced the smallest tumor among all the groups. Rate of tumor incidence is presented in Figure 7g; agomir-186 combined with shHIF-1α group resulted in 80% tumor incidence rate in both the MKN45 and SGC7901 groups; the tumor incidence rate in control groups were 100%. The *in vivo* study provided strong support for miR-186 on tumor suppression.

## Discussion

The current progression achieved in gastric cancer is still limited, mainly owing to the genetic complexity and heterogeneity. Adenocarcinoma accounts for the major part of the gastric cancer, and its metastasis emerges in an early stage. Searching for novel targets may bring a new opportunity.

The role of small non-coding RNAs, such as miRNAs, in oncogenesis is well documented. Studies show that miRNAs are frequently located in the cancer-associated genomic regions, including the loss of heterozygosity region, chromosome amplification region and fragile sites.^31, 32^ They are involved in many tumor biological processes. MiR-186 is aberrantly expressed in several cancers. Its downregulation correlates with poor prognosis in lung adenocarinoma and cell cycle.^8^ It also sensitizes ovarian cancer cells to cisplantin.^10^ Its overexpression attenuates lung cancer cell invasiveness through inhibiting PTTG1.^9^ MiR-186 therefore may be a candidate that link gastric adenocarcinoma to less aggressive phenotype. We first detected miR-186 abundance in HDAC and PDAC, and it represented a downward trend along with differentiation degree. According to cell viability assay and apoptotic assay in MKN45 and SGC7901 cells, introducing of miR-186 significantly inhibited cell proliferation and induced apoptosis. *In vivo* study confirmed the above results. We detected HIF-1α, the putative downstream target gene of miR-186 through bioinformatics prediction (http://www.microrna.org/microrna/home.do). The negative regulatory effect of miR-186 on HIF-1α was verified through miR-186 introduction and ablation. The luciferase reporter assay confirmed the direct binding of miR-186 to HIF-1α.

HIF-1 is located at 14q21–14q24. It is widely expressed in cancer center. Activations of hundreds of cellular transcriptional responses mediated by HIF-1 are common in cancers and usually confer poor prognosis.^33^ HIF-1α is the active subunit of HIF-1. Its C-terminal contains oxygen-dependent degradation domain and transactivation domain (TAD-C) that has a fine adjustment role and its N-terminal contains TAD-N that is essential for transcriptional activation. According to the previous reports,^34, 35^ HIF-1α regulates erythropoiesis-encoding gene, vascular endothelial growth factor, insulin-like growth factor II and glycolytic-related enzymes, including aldolase A and enolase 1. It affects erythropoiesis, angiogenesis, energy metabolism, cell survival and drug resistance to maintain tumor cells in a stable internal environment to adapt to hypoxia.^35, 36^

Aerobic glycolysis is one of the characteristic phenotypes of tumor cells and is the major way of energy supply. This phenomenon, also named the 'Warburg effect', is the most basic feature in tumorigenesis. Inhibition of the Warburg effect may be an effective tumor therapy strategy. Hypoxia can induce Warburg effect and HIF-1α may involve in the mechanism.^14^ The glycolytic markers, including glucose intake, lactic acid production, ATP and NADH production, were all upregulated when miR-186 was knocked down. Although the fast energy supply mode was attenuated by HIF-1α knockdown, the trends were consistent with gastric cancer cell survival. Nude mice xenograft assay also proved that miR-16 combined with shHIF-1α had produced the smallest tumor.

The three key enzymes of glycolysis are HK2, PFK-1 and pyruvate kinase. PFKP, as a major subtype of PFK-1 in cancer, catalyzes the second key step of glycolysis.^37^ According to our results, HIF-1α could bind to the promoter region and activate PFKP transcription. The process was supported by promoter Luciferase reporter assay and ChIP assay. PFKP as well as HK2 activities were also proved upregulated by HIF-1α overexpression.

HK2, as another downstream of HIF-1α in fermentation, participates in one irreversible step of glycolysis and acts as a rate-limiting enzyme. In multiple tumors, the overexpression of HK2 suggests a poor prognosis.^16, 38^ About 17–21% of gastric cancer patients were HK2 immunoreactivity positive.^39^ The present study showed that miR-186 regulated complex signaling cascade to have a role of glucose metabolism regulation via targeting HIF-1α. HIF-1α activated HK2 and accelerated energy supply from glycolysis; the degradation of HIF-1α by miR-186 could partly reverse the process and inhibit cell proliferation.

The PD-L1 also took part in HIF-1α-mediated gastric oncogenesis process according to the study. As a key signaling pathway in tumor immune escape, PD-L1 has a negative role in immune response regulation. Downregulated PD-L1 resulted in enhanced apoptosis and weakened survival of tumor cells.^40^ Attenuated glycolysis by HK2 and PFKP ablation may sensitize MNK45 and SGC7901 cells to apoptosis induced by PD-L1 blocking.^41, 42^ All these effects can be achieved through HIF-1α inhibition caused by miR-186 overexpression.

Our study supported that miR-186 was a hopeful novel target for gastric adenocarcinoma treatment. According to this result, some malignant tumor treatments, for instance, viral vector carrying miR-186 may be taken into consideration. Although practical clinical application is still in its infancy, non-coding RNA regulation of tumor growth in cancer treatment application still provides us a novel choice.

## Materials and methods

### Clinical specimens

Eighteen cases of normal GT, 17 cases of HDAC tissues and 16 cases of PDAC tissues were obtained from surgical resection in Shengjing Hospital of China Medical University. Normal GT were obtained from normal gastric perforation surgery. Tumor specimens were collected from gastric cancer radical surgery. Fresh tissues were sent for pathological diagnosis after surgical removal. The remaining parts were immediately stored in liquid nitrogen. All patients provided written informed consent. The experiment has been approved by the ethics committee of Shengjing Hospital of China Medical University.

### Cell culture

Gastric cancer cell lines MKN45 and SGC7901, human embryonic kidney cell line HEK-293T and human gastric mucosa cell line GES-1 were purchased from the Shanghai Institutes for Biological Sciences Cell Resource Center, Shanghai, China. MKN45 cells and HEK-293T cells were maintained in Dulbecco's modified Eagle's medium containing 10% fetal bovine serum (Life Technologies Corporation, Paisley, UK). SGC7901 and GES-1 cells were cultured in RPMI-1640 (Sigma-Aldrich, St Louis, MO, USA) containing 10% fetal bovine serum. All cells were incubated in a humidified incubator at 37 °C with 5% CO_2_.

### Cell transfection

MKN45 and SGC7901 cells were transfected with chemically synthesized agomir-186 and antagomir-186 (GenePharma, Shanghai, China). Human HIF-1α gene and short-hairpin RNA targeting HIF-1α gene were ligated into LV3-CMV-GFP-EF1a-Puro lentiviral vectors. LV3-CMV-GFP-Puro-HIF-1α, LV3-CMV-GFP-Puro-shHIF-1α and their respective empty vectors were transfected into HEK-293T cells with ViraPower Packaging Mix (Life Technologies Corporation). After 2 of days incubation, the virus-containing supernatant was collected to infect MKN45 and SGC7901 cells. The transfection efficiency was evaluated by qRT–PCR; the empty vector that was absent of target gene was used as a negative control. HIF-1α(+) and shHIF-1α cells were cotransfected with agomir-186 or antagomir-186 to the construct agomir-186+HIF-1α(+), agomir-186+shHIF-1α, antagomir-186+ HIF-1α(+) and antagomir-186+shHIF-1α groups.

### RNA extraction and qRT–PCR

MiR-186 abundance was measured using qRT–PCR. Total RNA was extracted using Trizol (Life Technologies Corporation). RNA concentration and quality were measured using NanoDrop spectrophotometer (ND-100, Thermo, Wilmington, DE, USA). TaqMan MicroRNA Reverse Transcription Kit (Applied Biosystems, Foster City, CA, USA) and TaqMan Universal Master Mix II (Applied Biosystems) were used for PCR amplification. U6 were used as endogenous controls for miRNA expression. A standard curve of threshold cycle (*C*_T_) value vs log input standard cDNA was constructed for each assay. Fold changes were calculated using the relative quantification (2^−^^Δ^^Δ^^Ct^) method.

### Western blotting

HIF-1α, PD-L1, HK2 and PFKP expression levels were detected by western blotting. Cell homogenate protein was prepared and boiled for 10 min (HIF-1α under normoxia could be degraded fleetly, the protein extraction process should be very quick). Samples were centrifuged at 17000 r.p.m., 4 °C for 25 min. The protein concentrations were measured using the BCA Protein Assay Kit (Beyotime Institute of Biotechnology, Shanghai, China).

The samples (40 μg each lane) that underwent electrophoresis in sodium dodecyl sulfate-polyacrylamide gel were transferred to polyvinylidene fluoride membrane. Membrane was blocked in Tween Tris-buffered saline buffer containing 5% bovine serum albumin and then incubated with primary antibodies, respectively, at 4 °C overnight. Primary antibodies include HIF-1α, PD-L1, HK2, PFKP (1:1000, Abcam, Cambridge, UK) and glyceraldehyde 3-phosphate dehydrogenase (1:5000, Cell Signaling, Beverly, MA, USA). Later, membranes containing protein bands were incubated with horseradish peroxidase-polymerization secondary antibodies for 2 h at room temperature. Detection of immune complexes went through ECL chemiluminescent detection system. The integrated density value was calculated with the software FluorChem2.0 (Alpha Innotech, San Leandro, CA, USA).

### CCK8 cell proliferation assay and clonogenic assay

MKN45 and SGC7901 cells were seeded in 96-well plates (Corning, Corning, NY, USA) at a density of 2000/well with five replicate wells each group. The ability of cell proliferation was detected using CCK8 reagent (Beyotime Institute of Biotechnology). Two days later, CCK8 reagent was added to the culture medium and incubated for 2 h, which was proved the most suitable time in the preliminary experiment. The investigator was blinded to the group allocator in the assay and other assays in the present study.

### Cell apoptosis assay

Cell apoptosis rate was measured with Annexin V-phycoerythrin (PE)/7-aminoactinomycin D (7-AAD) Kit (SouthernBiotech, Birmingham, AL, USA). Cells were stained with Annexin V-PE and 7-AAD for 15 min and then were subjected to flow cytometric (FACScan, BD, Biosciences, San Jose, CA, USA) detection. The apoptosis ratios were analyzed using the BD Accuri C6 software (BD Biosciences, Franklin Lakes, NJ, USA).

### Glucose uptake and lactate production detection

The glucose uptake assay was performed using the Glucose Test Kit (BioVision, Milpitas, CA, USA). MKN45 and SGC7901 cells were seeded in six-well plates at a density of 10^6^ per well at 37 °C for 48 h, and the medium at 0 h was collected as background glucose concentration. Glucose concentration reduction of medium was considered as cellular glucose uptake. Glucose uptake=(background concentration−reading concentration)/protein concentration.

For extracellular lactic acid production measurement, the cell culture medium was detected with Lactate Assay Kit (BioVision). The values were normalized to the protein concentration.

### Detection of ATP/ADP ratio

For the ATP/ADP ratio measurement, an ApoSENSOR ADP/ATP Ratio Assay Kit (BioVision) was used according to the manufacturer's instructions. Luminescence was measured using a spectrmax (Molecular Devices, Sunnyvale, CA, USA). Total number of 1 × 10^4^ cells were seeded into the luminometer plate and then Nucleotide Releasing Buffer was added. The ATP Monitoring Enzyme (1 μl) was added, and then the samples were read at 1 (data A) and 10 min (data B). Then the ADP Converting Enzyme was added, and the samples values were read (data C). ATP/ADP ratio=data A/(data C−data B).

### NAD+/NADH ratio assay

NADH and NAD+ were determined using EnzyLight ADP/ATP Ratio Assay Kit (Bioassay Systems, Hayward, CA, USA). For NAD+ determination, cell pellets were resuspended with 100 μl NAD+ extraction buffer. For NADH determination, 100 μl NADH extraction buffer was added. After heating at 60 °C for 5 min, 20 μl of assay buffer was added into the extracts. Then the mixtures were centrifuged at 13 000 r.p.m. for 5 min. Supernatants were transferred to working reagent and the optical densities at 565 nm were read at 0 and 15 min. Absorbance values were used to calculate NAD+/NADH ratios.

### PFK and HK2 activity test

PFK activity was assessed through the Phosphofructokinase Activity Colorimetric Assay Kit (Sigma, St Louis, MO, USA) according to the manufacturer's instructions. In the preexperiment, 30 min was proved to be the most suitable. HK2 activity was assessed through the Hexokinase Colorimetric Assay Kit (Sigma).

### Luciferase reporter assay

HIF-1α mRNA 3′UTR (untranslated regions) containing the predicted binding sites were selected. The theoretical binding sequence of miR-186 in HIF-1α gene and its mutant sequence was designed. The 3′UTR fragment of HIF-1α and its mutant were cloned into a firefly luciferase reporter construct pmirGLO Dual-luciferase vectors (GenePharma, Suzhou, China), respectively. HEK 293T cells were cotransfected with HIF-1α-3′UTR-Wt (or HIF-1α-3′UTR-Mut) and agomir (or agomir-186-NC). After transfection, cells were harvested, lysed and subjected for the dual luciferase reporter assay system (Molecular Devices).

By screening the promoter region of PFKP, a binding motif (CACGC) of HIF-1α was discovered. To determine the responsive HIF-1α-binding sites in the human PFKP promoter, promoter activities were measured using Dual-Luciferase Reporter Assay System. Human full-length HIF-1α sequence was cloned into pEX3 vector (GenePharma). pEX3-HIF-1α and its empty vector were transfected into MKN45 and SGC7901 cells. Renilla promoters were cotransfected as an internal control.

### Chromatin immunoprecipitation

ChIP was conducted using the Simple ChIP Enzymatic Chromatin IP Kit (Cell Signaling Technology, Danvers, MA, USA). Cells were cross-linked with 1% paraformaldehyde and collected in lysis buffer containing phenylmethanesulfonyl fluoride. Chromatin was cleaved into fragments of nucleic acids (200–1000 bp). The 1000-bp upstream region of the putative HIF-1α-binding site was used as a negative control. Lysates of 2% concentration were used as an input control; the remaining lysates were immunoprecipitated with normal mouse IgG and HIF-1α antibody.

DNA was extracted and amplified by PCR. The forward and reverse primers were: 5′-AGGGCGTCTCTGAGGGTC-3′ and 5′-AGGTGGTGGGAAAAGGTGA-3′.

### Subcutaneous xenograft model in nude mice

For the *in vivo* study, the stably overexpressed miR-186 cells, HIF-1α stable knockdown cells and agomir-186 combined with shHIF-1α cells were collected. Human HIF-1α shRNA was ligated into pGPU6/GFP/Neo vector to construct shHIF-1α plasmid. After transfection with shHIF-1α plasmid, MKN45 and SGC7901 cells were selected using Geneticin (G418; Sigma-Aldrich) to construct the stable shHIF-1α cell lines. MiR-186 sequence was cloned into pLenti6.3/V5-DEST vector to construct PLenti6.3/V5-DEST-agomir-186 lentiviral vector. Cells stably expressing shHIF-1α were then lentivirally transfected with agomir-186 sequence to create agomir-186+shHIF-1α stable cells for nude mice xenograft assay.

Male nude mice (balb/c) aged 4 weeks were raised under specific pathogen-free condition. All process involving animals were subjected to approval by the Research Animal Care and Use Committee of Shengjing Hospital. Cells were subcutaneously implanted into the right flanks of mice, at a density of 5 × 10^5^ cells. Each group has 10 mice. Mice that died within 7 days postoperation were considered unrelated with tumor and eliminated from the data; new mice were raised and supplemented to the groups. The investigator was blinded to the group allocator. Tumor volumes were monitored every 5 days, until 50 days postinoculation. Tumor volume (mm^3^)=length × width^2^/2.

### Fluorescent in situ hybridization of the tissues

Fresh normal GT and gastric cancer, tissues were fixed with paraformaldehyde with 1/1000 diethypyrocarbonate, embedded, cut into slices (6 μm), dewaxed, rehydrated in graded ethanol and soaked in the 0.3% hydrogen peroxide. Slides were then digested and hybridized with the miR-186 Hybridization Assay Kit (BOSTER, Wuhan, China). Slides were incubated with biotinylated antibody and then SABC-FITC (strept actividin–biotin complex–fluorescein isothiocyanate) following the manufacturer's instructions. Sections were imaged under a microscope (Olympus, Tokyo, Japan) at × 400 magnification; the investigator was blinded to the group allocator.

### Immunohistochemistry assays

Specimens were fixed, embedded and sliced into slides (4-μm thick). Slides were then dewaxed, rehydrated and incubated in 0.3% H_2_O_2_. After antigen epitope repairing, sections were blocked with 10% normal goat serum (BOSTER) and then with antibody against HIF-1α (1:100; Abcam). Slides were incubated with respective biotinylated IgG. Samples were stained with 3,3'-diaminobenzidine. Sections were imaged under a light microscope (Olympus) at × 400 magnification.

### Statistical analysis

All data were derived from at least three independent experiments. Statistical analyses were performed with Student's *t*-test using the SPSS 18.0 statistical software (IBM, New York, NY, USA). The results were displayed as means±s.d. It was considered statistically significant when *P*<0.05.

## Conclusion

MiR-186 is lowly expressed acts as a tumor-suppressor gene in gastric cancer. It binds to the 3′UTR of pro-oncogenic gene HIF-1α in a base-pairing manner and inhibits its protein production. Ectopic expression of miR-186 suppresses proliferation and glycolytic metabolism of gastric cancer cells mainly by HIF-1α. Our study has shed a light for the development of non-coding-RNA-directed diagnostics and therapeutics against gastric cancer.
