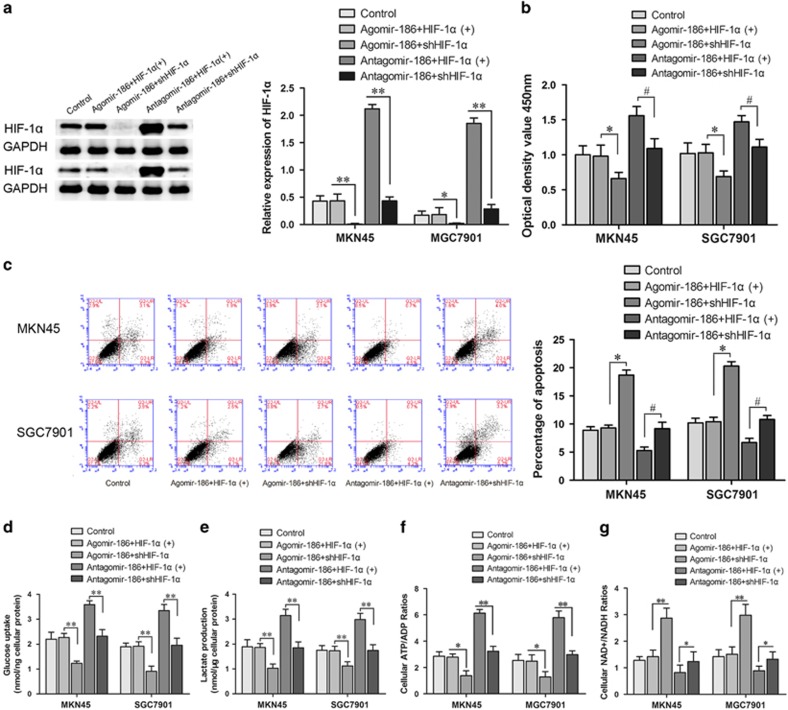# MiR-186 inhibited aerobic glycolysis in gastric cancer via HIF-1α regulation

**DOI:** 10.1038/oncsis.2017.20

**Published:** 2017-04-17

**Authors:** L Liu, Y Wang, R Bai, K Yang, Z Tian

**Correction to:** *Oncogenesis* (2016) **5**, e224; doi:10.1038/oncsis.2016.35; published online 9 May 2016

Since the publication of the above article, the authors have realized that both Figure 2c, apoptosis figure of Agomir-186-NC group of SGC7901 cell, and Figure 4c, apoptosis figure of Antagomir-186+shHIF-1α, were wrongly used.

The corrected figures are shown here.

The authors would like to apologize to the readers for any inconvenience this may have caused.

## Figures and Tables

**Figure 2:**
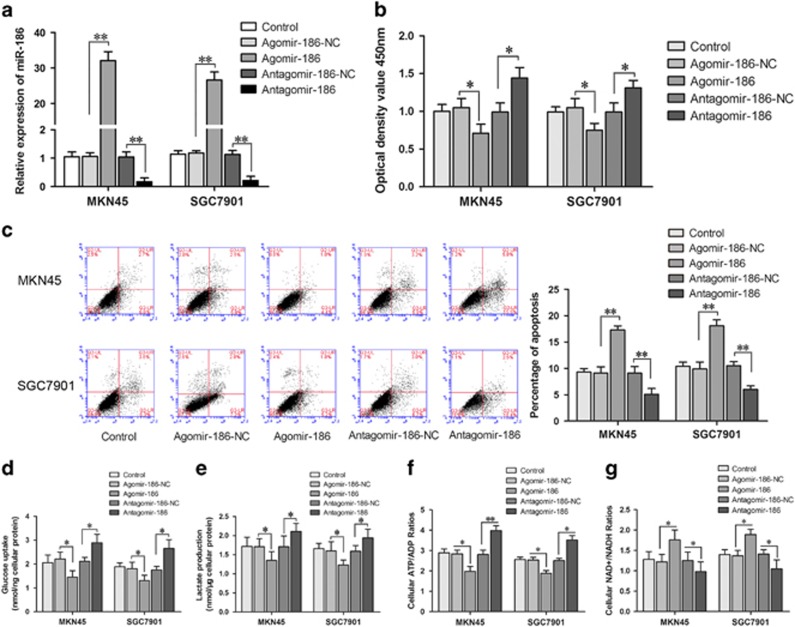


**Figure 4:**